# Use of a Reflectance Spectroscopy Accessory for Optical Characterization of ZnO-Bi_2_O_3_-TiO_2_ Ceramics

**DOI:** 10.3390/ijms12031496

**Published:** 2011-02-25

**Authors:** Mohd Sabri Mohd Ghazali, Azmi Zakaria, Zahid Rizwan, Halimah Mohamed Kamari, Mansor Hashim, Mohd Hafiz Mohd Zaid, Reza Zamiri

**Affiliations:** 1 Department of Physics, Faculty of Science, Universiti Putra Malaysia, 43400 UPM Serdang, Selangor, Malaysia; E-Mails: mgm.sabri@gmail.com (M.S.M.G.); zahidrizwan64@gmail.com (Z.R.); halimah@science.upm.edu.my (H.M.K.); mansor@science.upm.edu.my (M.H.); mhmzaid@gmail.com (M.H.M.Z.); zamiri.r@gmail.com (R.Z.); 2 Advanced Materials and Nanotechnology Laboratory, Institute of Advanced Technology, Universiti Putra Malaysia, 43400 UPM Serdang, Selangor, Malaysia

**Keywords:** UV-Vis spectrophotometer, Reflectance Spectroscopy Accessory, optical band-gap, ZnO, Bi_2_O_3_, TiO_2_

## Abstract

The optical band-gap energy (*E*_g_) is an important feature of semiconductors which determines their applications in optoelectronics. Therefore, it is necessary to investigate the electronic states of ceramic ZnO and the effect of doped impurities under different processing conditions. *E*_g_ of the ceramic ZnO + *x*Bi_2_O_3_ + *x*TiO_2_, where *x* = 0.5 mol%, was determined using a UV-Vis spectrophotometer attached to a Reflectance Spectroscopy Accessory for powdered samples. The samples was prepared using the solid-state route and sintered at temperatures from 1140 to 1260 °C for 45 and 90 minutes. *E*_g_ was observed to decrease with an increase of sintering temperature. XRD analysis indicated hexagonal ZnO and few small peaks of intergranular layers of secondary phases. The relative density of the sintered ceramics decreased and the average grain size increased with the increase of sintering temperature.

## 1. Introduction

Polycrystalline zinc oxide (ZnO) is used extensively in rubber, paint, cosmetics and textile industries as well as in the electronic industry. The ZnO based varistor is widely used as an electronic component in automobile electronics and also in sophisticated semiconductor electronics. ZnO based varistors are fabricated with different type of additives which play an important role in its non-linear characteristics. Its unique grain boundary feature is responsible for non-linear current-voltage (*I*–*V*) characteristics of the device [1,2] and thus is used to protect electrical equipment against unwanted electrical surges. Currently, ZnO based varistors are being used for low-voltage applications. ZnO based varistors are fabricated with small amounts of different metal oxides such as Bi_2_O_3_, CoO, MnO, Sb_2_O_3_, and TiO_2_ [3–8]. TiO_2_ also acts as an active photocatalyst and is discussed further by Linsebigler *et al.* [9]. *I*–*V* studies have been extensively investigated for the ZnO based varistor by previous researchers [5,10] and it is necessary to investigate the electronic states of ceramic ZnO and the effect of doped impurities with different processing conditions.

The measurement of the absorption spectrum in semiconductors leads to the determination of the optical band-gap energy [11,12]. In solid state technique, powdered samples are usually produced instead of thick or thin films. Thus, commonly UV-Vis spectroscopy is carried out by dispersing the powdered samples in solutions like deionized water, acetone, ethanol or other alcohols. One of the major problems is that samples often precipitate due to the particle size not being small enough, making the absorption spectrum difficult to analyze. In order to avoid these consequences, it is preferable to use a Reflectance Spectroscopy Accessory (RSA), which reliably obtains the optical band gap of powder samples. In similar work, Morales *et al.* [13] used diffuse reflectance spectroscopy for powdered nanostructures for optical property measurements.

In this study, investigation regarding the optical properties of powdered of ZnO doped with Bi_2_O_3_ and TiO_2_ at different sintering temperatures and times are discussed by using a UV-Vis Spectrophotometer attached to a RSA.

## 2. Experimental

Oxide precursors of 99.9% purity (Alfa Aesar) were used. The composition consists of 99 mol% ZnO + 0.5 mol% Bi_2_O_3_ + 0.5 mol% TiO_2_ powder. The powder was ball milled for 24 hours in deionized water. The slurry was dried at 70 °C using a hot plate. It was continuously magnetically stirred to avoid sedimentation of the heavy particles and pre-sintered at 800 °C for two hours. The pre-sintered mixture was pulverized using an agate mortar/pestle and after addition of 1.75 wt% Polyvinyl Alcohol (PVA) binder, was granulated by sieving through a 75 micron mesh screen. The mixture then was pressed into discs of 10 mm in diameter and 1 mm in thickness, each at a pressure of 2 ton/m^2^. Finally, the discs were sintered at 1140, 1170, 1200, 1230 and 1260 °C with 45 and 90 minutes sintering duration at a heating and cooling rate of 2.66 °C min^−1^. The disk from each sample was ground for optical and XRD characterization.

The crystalline phases were identified by an XRD (PANalytical X’Pert Pro PW3040/60, Philips) with CuKα radiation and the data were analyzed using X’Pert High Score software. The density was measured by the geometrical method [14]. For the microstructure analysis, each disk sample was thermally etched at 150 °C in a tube furnace.

The microstructure was examined by Variable Pressure Scanning Electron Microscopy (VPSEM, Leo 1455). The average grain size (*d*) was determined by lineal intercept method [15], given by:

(1) d=1.56L/MN

where *L* is the random line length on the micrograph, *M* is the magnification of the micrograph and *N* is the number of the grain boundaries intercepted by lines.

The UV-Vis Spectrophotometer (Lambda 35, Perkin Elmer) was used to measure the optical band-gap energy of the ceramics. The transmission signal was measured for the wavelength from 200 to 800 nm and then converted to absorption signal for further evaluation [16]. It was assumed that the fundamental absorption edge of the ceramics is due to the direct allowed transition. The optical band-gap energy is given by [17]:

(2) $${\left(Ah\upsilon \right)}^{2}=C(h\upsilon -E_{g})$$

near the optical band-gap, where *A* is the optical absorption coefficient, *C* is the constant independent of photon energy (*hυ*), and *E_g_* is the direct allowed optical band-gap energy. From the plot of (*Ahυ*)^2^ *versus hυ*, the value of *E_g_* is obtained by extrapolating the linear fitted regions to (*Ahυ*)^2^ = 0.

## 3. Results and Discussion

**Figure 1.**
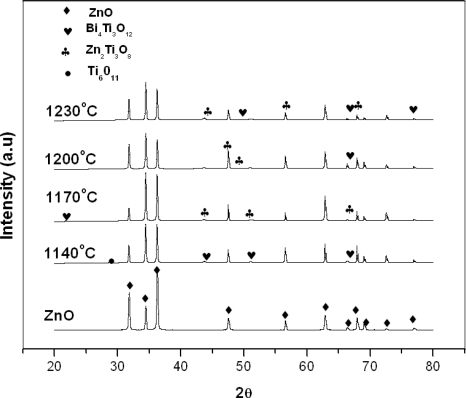
XRD patterns of ZnO based varistor at different sintering temperatures with a sintering time of 45 minutes.

The XRD analysis, Figure 1, reveals diffraction peaks which belong to two phases, *i.e.*, ZnO (ICSD code: 067454) and intergranular layers in the varistor ceramics. The intergranular layers are composed of Ti_6_O_11_ and appear as a very small peak in the XRD pattern for the sample sintered at 1140 °C for 45 minutes sintering time only due to removal of oxygen from TiO_2_ in a solid state reaction [9]. Many secondary phases with small peaks were detected in the ceramics, namely, Bi_4_Ti_3_O_12_ (ICSD code: 024735) and Zn_2_Ti_3_O_8_ (ICSD code: 022381) at all sintering temperatures. Sung and Kim [18] and Suzuki and Bradt [19] have suggested that below 1030 °C, TiO_2_ will dissolves in the Bi_2_O_3_-rich liquid phase, reacting with the Bi_2_O_3_ liquid and forming the compound Bi_4_Ti_3_O_12_, due to the following reaction, but with few modifications for suitable equilibrium reaction as followswith XRD pattern analysis,

(3) $$2{\text{Bi}}_{2}{\text{O}}_{3}\;(\text{l})+3{\text{TiO}}_{2}\;(\text{s})\to {\text{Bi}}_{4}{\text{Ti}}_{3}{\text{O}}_{12}\;(\text{s})$$

The solid Bi_4_Ti_3_O_12_ is then reported to decompose and react with the solid ZnO grains at about 1050 °C, according to the reaction [17,18],

(4) $${\text{Bi}}_{4}{\text{Ti}}_{3}{\text{O}}_{12}\;(\text{s})+6\text{ZnO}\;(\text{s})\to 2{\text{Bi}}_{2}{\text{O}}_{3}\;(\text{l})+3{\text{Zn}}_{2}{\text{TiO}}_{4}\;(\text{s})$$

Due to the appearance of Zn_2_Ti_3_O_8_ at high temperatures, we suggest the following reaction may occur,

(5) $${\text{Bi}}_{4}{\text{Ti}}_{3}{\text{O}}_{12}\;(\text{s})+2\text{ZnO}\;(\text{s})\to 2{\text{Bi}}_{2}{\text{O}}_{3}\;(\text{l})+{\text{Zn}}_{2}{\text{Ti}}_{3}{\text{O}}_{8}\;(\text{s})$$

Both of the Bi_4_Ti_3_O_12_ and Zn_2_Ti_3_O_8_ phases are revealed to coexist in the sample sintered at all sintering temperatures except 1140 °C for a sintering time of 45 minutes that only presents the Bi_4_Ti_3_O_12_ phase as shown in Figure 1.

**Figure 2.**
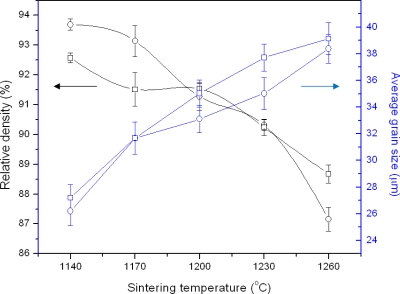
Relative density and average grain size of ZnO based varistor at different sintering temperatures for (□) 45 minutes and (○) 90 minutes sintering time.

**Figure 3.**
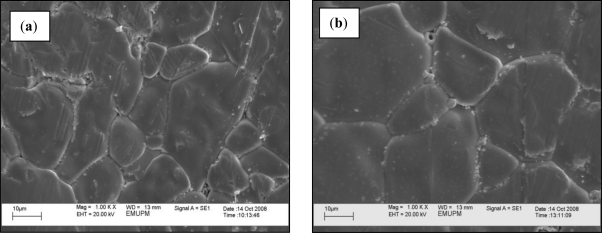
Scanning electron micrographs of ceramics after sintering at (**a**) 1170 °C and (**b**) 1260 °C.

The relative density of sintered ceramics decreased from 93.70 to 87.16% of theoretical density (5.67 g/cm^3^) with the increase of sintering temperature, Figure 2. The average grain size increased from 26.2 to 38.4 micron with the increase of sintering temperature, Figure 2. The increase in average grain size, observed with both sintering durations, is due to the TiO_2_ which is a strong grain enhancer. It was observed under VPSEM that a few abnormal grains of irregular shape and size were distributed in the whole sample especially at high sintering temperature. The abnormal grain growth increased with increase of sintering temperature, Figure 3. This indicates that the pores increase with the increase of sintering temperature. The pores are trapped between the large grains in the ceramics at high sintering temperature [20].

**Figure 4.**
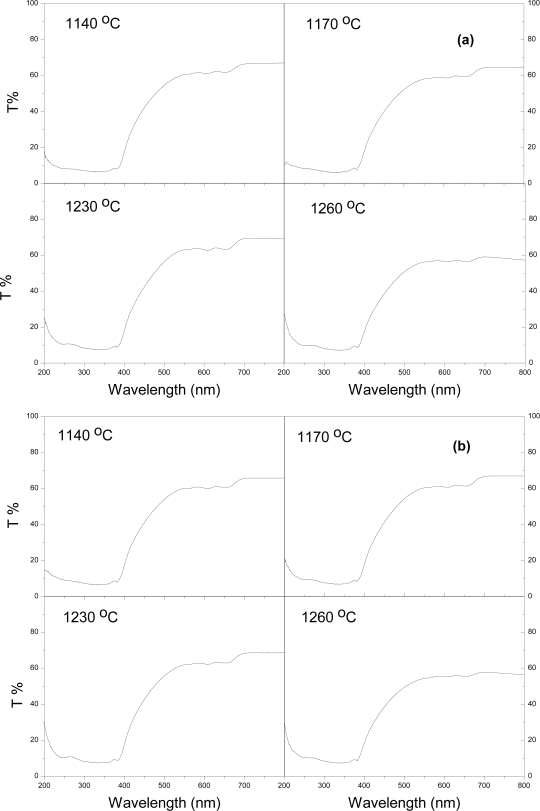
Transmission spectra of ZnO-Bi_2_O_3_-TiO_2_ ceramics after (**a**) 45 minutes and (**b**) 90 minutes sintering time.

**Figure 5.**
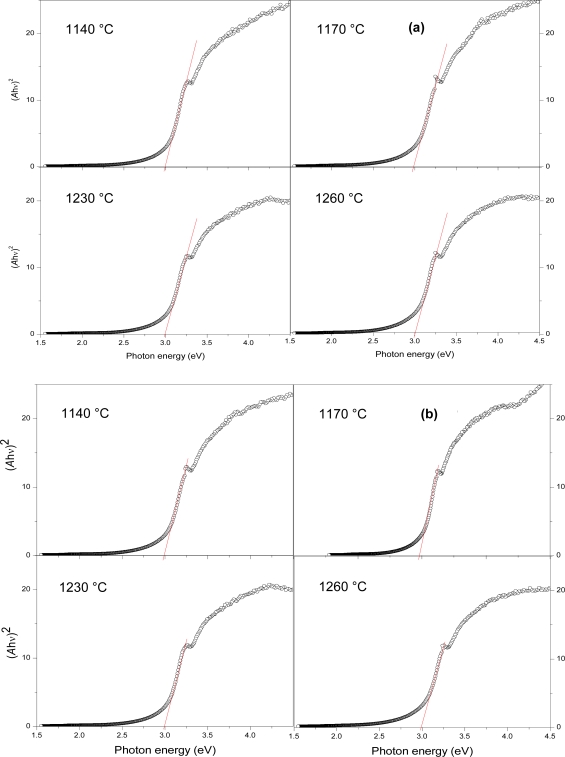
Transformed spectra of ceramics of (**a**) 45 minutes and (**b**) 90 minutes sintering time.

**Figure 6.**
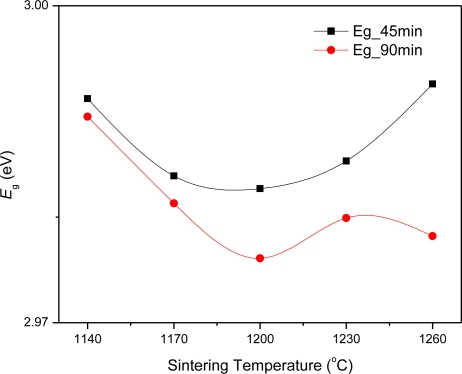
The *E*_g_ of ceramics of (□) 45 minutes and (○) 90 minutes sintering time at different sintering temperatures.

From the raw data of *T*% against wavelength (Figure 4), *E_g_* is obtained through Equation 2, (*Ahυ*)^2^ against *hυ*, Figure 5. *E_g_* values are 2.99, 2.98 eV, at the sintering temperatures 1140, 1230 °C, respectively, for 45 minutes sintering time, and slightly decrease with 90 minutes sintering, Figure 6. It is observed that the trend of *E_g_* is constant with a 45 minute sintering time. The liquid phase of Bi_2_O_3_ generates the interface state which reduces the *E_g_* of pure ZnO [21]. Doping TiO_2_ in ZnO-Bi_2_O_3_ system slightly reduces the *E_g_*. Only at a sintering temperature of 1140 °C shows the limited substitution of Ti^4+^ ions in the ZnO lattice as the ionic radii of Ti^4+^ (0.68 Å) smaller than that of Zn^2+^ (0.74 Å). At other higher temperatures, additional interface states are generated that reduce the *E_g_*. This reduction of *E_g_* correlates with the structural disordering increment of Bi_4_Ti_3_O_12_ in the grain boundaries with the increase of sintering temperature. For 90 minutes sintering time, the *E_g_* values slightly decrease from 1140 to 1260 °C, which might be due to growth of interface states at the surface of the particles and at the grain boundaries.

## 4. Conclusions

The UV-Vis spectrophotometer results of ZnO ceramic with variations in sintering temperature proved the decrease of *E_g_* with an increase of sintering temperature and time, showing the segregation of Bi_2_O_3_ at grain boundaries and possible substitution of Ti ion with Zn ion, which creates interface states. These results are correlated with the analysis obtained by XRD which shows a structural disordering in the grain boundaries.
